# Why are biting flies attracted to blue objects?

**DOI:** 10.1098/rspb.2023.0463

**Published:** 2023-06-28

**Authors:** Roger D. Santer, Otar Akanyeti, John A. Endler, Ismael Galván, Michael N. Okal

**Affiliations:** ^1^ Department of Life Sciences, Aberystwyth University, Aberystwyth SY23 3FG, UK; ^2^ Department of Computer Science, Aberystwyth University, Aberystwyth SY23 3DB, UK; ^3^ Centre for Integrative Ecology, School of Life and Environmental Sciences, Deakin University, Waurn Ponds, Victoria 3216, Australia; ^4^ Department of Evolutionary Ecology, National Museum of Natural Sciences, CSIC, 28006 Madrid, Spain; ^5^ International Centre of Insect Physiology and Ecology, PO Box 30772-00100, Nairobi, Kenya

**Keywords:** colour vision, vector control, host seeking, tsetse, stable fly, horse fly

## Abstract

Diurnal biting flies are strongly attracted to blue objects. This behaviour is widely exploited for fly control, but its functional significance is debated. It is hypothesized that blue objects resemble animal hosts; blue surfaces resemble shaded resting places; and blue attraction is a by-product of attraction to polarized light. We computed the fly photoreceptor signals elicited by a large sample of leaf and animal integument reflectance spectra, viewed under open/cloudy illumination and under woodland shade. We then trained artificial neural networks (ANNs) to distinguish animals from leaf backgrounds, and shaded from unshaded surfaces, in order to find the optimal means of doing so based upon the sensory information available to a fly. After training, we challenged ANNs to classify blue objects used in fly control. Trained ANNs could make both discriminations with high accuracy. They discriminated animals from leaves based upon blue–green photoreceptor opponency and commonly misclassified blue objects as animals. Meanwhile, they discriminated shaded from unshaded stimuli using achromatic cues and never misclassified blue objects as shaded. We conclude that blue–green opponency is the most effective means of discriminating animals from leaf backgrounds using a fly's sensory information, and that blue objects resemble animal hosts through such mechanisms.

## 1. Background

Attraction to blue objects is virtually universal among diurnal biting flies, including the Glossinidae (tsetse), Hippoboscidae (keds), Muscidae (e.g. stable flies), Tabanidae (horse or march flies) and Simuliidae (blackflies) [1–7]. This well-documented behaviour is exploited by a variety of blue traps and insecticide-treated targets developed to control the flies and the diseases they spread [8–13]. However, the functional significance of attraction to blue for the flies themselves remains unclear, particularly since blue objects are relatively rare in the natural environment.

Three competing hypotheses have been proposed to explain the attraction of biting flies to blue objects, namely that blue objects resemble animal hosts [1,14]; blue surfaces resemble shaded resting places [15,16]; and blue attraction is a by-product of attraction to polarized light [17]. First, in terrestrial habitats containing plants, the reflectance spectra of natural surfaces fall into three classes—(i) those of green leaves with a reflectance peak at approximately 555 nm (termed ‘leaf green’); (ii) most inorganic and many organic surfaces, including melanin-pigmented animal integuments, for which reflectance monotonically increases with wavelength (here termed ‘grey–brown’); and (iii) those of signalling colours such as fruits, flowers and ornaments that follow no particular pattern but contrast against a leaf background (termed ‘leaf contrast’) [18]. In such environments, a host-seeking biting fly must detect grey–brown spectra among a predominantly leaf green background, and it has been proposed that blue stimuli contrast against such backgrounds and activate the perceptual mechanisms that make this classification [1,14]. Second, the shade that occurs under open canopies, or isolated trees and other objects (termed ‘woodland shade’) is relatively richer in shorter wavelengths of light compared to direct sunlight, and thus these kinds of shadows appear blueish [19]. On this basis, it has been suggested that blue objects are attractive to biting flies such as tsetse because they attend to the blueness and darkness of shadows to locate cover such as tree bark fissures and rot holes in which they frequently rest [15,16]. Finally, because some blue- and UV-sensitive photoreceptor types of tabanid flies have maximum sensitivity to linearly polarized light in opposing planes, the flies' perceptions of colour and polarization are confounded [17]. On this basis, it has been suggested that the blue preference of these and other biting flies is a by-product of a polarization sensing mechanism that the flies are proposed to use to identify bodies of water and to segregate potential hosts from background [17].

**Figure 1.**
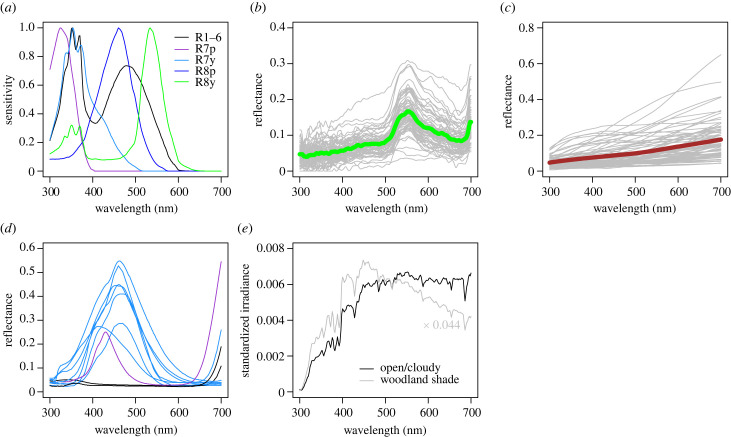
Input spectra for photoreceptor excitation calculations. (*a*) Flies possess five spectral classes of photoreceptor through the majority of their compound eyes, and the responses of these photoreceptors provide the inputs to visually guided behaviours. Plot shows sensitivity functions for the photoreceptors of *Musca* and *Calliphora*. Data were published in [20], copyright Elsevier, and are as used by [21]. Excitations for each of these photoreceptors were calculated for samples of leaf and animal stimuli, and for blue, black and violet surfaces of biting fly control devices. (*b*) The sample of 72 leaf reflectance spectra used as stimuli in our analysis, with the mean leaf reflectance spectrum plotted as a thick green line. Data are from the Floral Reflectance Database [22]. (*c*) The sample of 72 animal reflectance spectra used as stimuli in our analysis, with the mean animal reflectance spectrum plotted as a thick brown line. Data are from [23]. (*d*) The sample of 11 blue, black and violet (purple to a human eye) surfaces from biting fly control devices. Data were published in [3–5,24]. (*e*) Photoreceptor excitations for each stimulus were calculated under both open/cloudy and woodland shade irradiance spectra. These were the mean spectra from [19] and are shown standardized so that photon flux density values sum to 1. The spectra differed greatly in total intensity, and the total intensity of the woodland shade spectrum was 4.4% of that for the open/cloudy spectrum. Further details on spectra are available at [25].

In attempting to differentiate these hypotheses, it is essential to consider how these stimuli are processed by the perceptual mechanisms of a fly, which differ greatly from those of humans. The visual systems of calyptrate flies have been most thoroughly investigated in the housefly *Musca* and blowfly *Calliphora*, and these flies possess five spectral classes of photoreceptor across the majority of the compound eye (excluding areas specialized for tracking mates and receiving polarized light; figure 1*a*) [20]. In these flies, each ommatidium has eight photoreceptors named R1–R8. R1–6 are similar in every ommatidium of the eye and contain a photopigment with peak sensitivity (*λ*_max_) at *ca* 490 nm, and three sensitivity peaks at approximately 332, 350 and 369 nm due to a sensitizing pigment [20,26]. The rhabdomeres of the R7 and R8 photoreceptors are stacked centrally within each ommatidium. In about 30% of ommatidia the ‘p’ (pale) form occurs, in which R7p possesses a UV-sensitive photopigment (*λ*_max_ *ca* 335 nm), and R8p a blue-sensitive photopigment (*λ*_max_ *ca* 460 nm) [20]. In the other 70% of ommatidia the ‘y’ (yellow) form occurs, in which R7y has sensitivity peaks at 337, 355 and 373 nm due to a sensitizing pigment, since its rhabdomere appears to contain both a blue-sensitive photopigment and a carotenoid screening pigment [20,27]. Meanwhile, R8y contains a photopigment sensitive to green wavelengths (*λ*_max_ *ca* 520 nm), but also contains a UV-sensitive sensitizing pigment and experiences screening from the overlying R7y rhabdomere [20,27,28]. Although other calyptrate flies have been less intensively studied, broadly the same basic arrangement appears to exist. For example, genomic analyses show that *Stomoxys calcitrans* and several tsetse (*Glossina* spp.) possess the same opsin types [29,30], and although electrophysiological characterization of tsetse photoreceptors is not complete, the same classes of photoreceptor seem to be present with only an approximately 10 nm increase in the *λ*_max_ of the R1–6 photopigment [31]. Outside of the Calyptratae, slightly more variation in the sensitivity of these five spectral classes is evident. In *Drosophila*, the UV-sensitivity of the R7y photoreceptor is conferred by an opsin rather than a sensitizing pigment [32]. In the horsefly *Tabanus bromius*, electrophysiological recordings indicate that the *λ*_max_ of the R1–6 photopigment is shifted to longer wavelengths compared to *Musca* and *Calliphora*, the spectral sensitivities of the UV-sensitive photoreceptors analogous to R7p and R7y are similar to each other, and the photoreceptor analogous to R8p appears to have an additional sensitivity peak in the UV [17]. Nevertheless, the complement of photoreceptors in the retinae of these higher flies is broadly similar.

Visual perceptions are formed by the neural processing of photoreceptor signals. Where these mechanisms involve the comparison of photoreceptor responses they are said to be chromatic, corresponding to the qualities of hue and saturation in human vision [33]. Where the mechanisms involve single photoreceptor responses, or sums of photoreceptor responses, they are said to be achromatic, corresponding to brightness in human vision [33]. Our understanding of the precise ways in which fly photoreceptor signals are processed to create perceptions is still developing. The R7 and R8 photoreceptors are believed to serve colour vision, and the prevailing view, based on behavioural work on the blowfly *Lucilia*, has been that their signals are processed via two colour-opponent channels, each involving a comparison of the R7 and R8 photoreceptor signals of a given ommatidial type [34,35]. However, although the R1–6 photoreceptors provide an achromatic channel serving motion vision, it has now been shown that these photoreceptors also contribute to colour vision in *Drosophila* [36].

In this study, we test the hypotheses that blue objects resemble animal hosts, and that blue objects resemble shaded resting places, in terms of the sensory information available to a fly. We also investigate whether the mechanisms that can best classify these stimuli rely on chromatic or achromatic processing of fly photoreceptor signals. We trained artificial neural networks (ANNs) to identify potential hosts, or resting places in woodland shade, using only the five photoreceptor signals that would be available to a typical fly. The process of training an ANN optimizes the weights of connections between a network of artificial neurons, meaning that stimuli could be classified based upon chromatic or achromatic mechanisms in a manner analogous to real nervous systems, free from assumptions about how photoreceptor signals should be processed. Used in this way, ANNs can be considered abstract mechanistic models of the fly nervous system, replicating input–output relationships and providing a practical working hypothesis regarding mechanisms [37]. We do not expect that ANNs will exactly mimic real fly nervous systems, but we anticipate that they will capture key features of their processing because evolution should also drive towards optimality in response to the selective pressures imposed by behaviour. After training, we challenged ANNs with the photoreceptor signals that would result from blue objects used for fly control, investigating whether ANNs optimized to solve natural classification problems using the sensory information available to a fly would misclassify those artificial blue stimuli as animal hosts or resting places in woodland shade.

## 2. Methods

### (a) Reflectance spectra

We focused our analysis on two out of the three main classes of natural spectra [18] that we considered to be relevant to host-seeking by biting flies: (i) ‘leaf green’ spectra (i.e. foliage) and (ii) ‘grey–brown’ spectra (i.e. melanin-pigmented animal integuments); we ignored ‘leaf contrast’ spectra (i.e. signalling colours) since such spectra follow no particular pattern and are relatively rare in natural visual environments. Our approach was to represent the variability within the chosen spectral categories, and not to characterize the exact spectra that any particular species of biting fly would experience. To represent a diversity of leaf green spectra, we assembled a database of 72 leaf reflectance spectra for different plant species randomly sampled from the Floral Reflectance Database (www.reflectance.co.uk) [22], based upon their accession numbers. These spectra were subsampled for 2 nm wavelength resolution (figure 1*b*). To represent a diversity of grey–brown spectra, we focused on the melanin-based coloration of animals. We obtained 72 reflectance spectra that were originally collected to sample the diversity of melanin (including eumelanin and pheomelanin)-based coloration in animals [23]. These spectra were for the feathers and hair of 58 species of bird (59 spectra) and 12 species of mammal (13 spectra), including colour patches that are black, grey, brown and orange to the human eye [23] (figure 1*c*). Because such spectra have a characteristic shape that depends on their melanin chemical composition [23], the sample covered a diversity of colorations rather than taxonomic groups. The shapes of these spectra resemble those measured for biting fly hosts such as cattle and deer [38]. These spectra were available at 10 nm wavelength resolution, and we linearly interpolated them to achieve equivalent resolution to the leaf spectra. This choice was of no great importance since photoreceptor excitation values calculated as below from spectra with 2 nm and 10 nm resolution were perfectly correlated (Spearman's rank correlation, *R*_s_ = 1.000, *n* = 288, *p* < 0.001). In addition, we obtained 11 spectra for effective biting fly control devices, comprising blue but also black and violet fabrics [3–5,24] (figure 1*d*). Further details on these spectra are available at [25].

### (b) Calculating photoreceptor signals

Photoreceptor responses and not reflectance spectra provide the inputs to visually guided behaviour, so we next calculated the responses in each of a fly's five main classes of photoreceptor to the stimuli described above (cf. [21,33,39]). We wanted to consider a hypothetical, generic fly in an open area viewing a stand of vegetation and visually searching it for a potential host or a shaded resting place. We thus performed these calculations twice: first considering each stimulus under open/cloudy (white) illumination as if in an unshaded position among the vegetation, and then considering each stimulus under woodland shade illumination (blue–grey) as if in a shaded position [19]. The quantum catch, *Q*, for each photoreceptor type was calculated as follows:

$$Q=\frac{\int_{300}^{700}I_{s}(\lambda )\cdot R_{s}(\lambda )\cdot S_{i}(\lambda )\;d\lambda }{\int_{300}^{700}I_{b}(\lambda )\cdot R_{b}(\lambda )\cdot S_{i}(\lambda )\;d\lambda },$$

where *I_s_* and *I_b_* are the irradiance spectra on the stimulus and background, respectively, *R_s_* and *R_b_* are the reflectance spectra of the stimulus and predominant background, and *S* is the photoreceptor sensitivity spectrum for receptor type *i*. The numerator represents the quantum catch resulting from a stimulus of interest, and the denominator adjusts that response according to the background stimulation, representing the process of adaptation by photoreceptors (cf. [39]). Since the predominant background during the search of a vegetation stand is considered to be leaves, *R_b_* was the mean reflectance spectrum calculated across our sample of 72 leaves (figure 1*b*). In all calculations, *I_b_* was the mean open/cloudy irradiance spectrum recorded by [19] (figure 1*e*). When carrying out these calculations for stimuli in unshaded positions, *I_s_* was the same mean open/cloudy irradiance spectrum, and for stimuli in shaded positions, *I_s_* was the mean woodland shade irradiance spectrum recorded by [19] (figure 1*e*). Irradiance spectra had units of photon flux, since energy units are irrelevant to studies of vision [40]. *S_i_* were the photoreceptor sensitivity spectra characterized for *Musca* and *Calliphora*, originally obtained from [20] and as used by [21] (figure 1*a*). This choice was justified because photoreceptor sensitivity spectra are most thoroughly characterized in these species, the spectral sensitivities of other higher fly species appear to be broadly similar (see §1) and the Muscidae include the obligate, cosmopolitan blood feeders of the genus *Stomoxys*.

Since photoreceptor responses relate nonlinearly to the number of quanta absorbed, we calculated values to represent photoreceptor excitation, *E*, as follows (cf. [41]):

E=ln(Q).

In total, our database contained *E* values for a fly's five main classes of photoreceptor for a total of 288 stimuli comprising 72 leaves and 72 animal integuments, each viewed under open/cloudy and woodland shade illumination.

To check the importance of assumptions, we repeated our work using alternatives to the nonlinearization step above and calculating *E* values using photoreceptor sensitivities recorded for the tsetse *Glossina morsitans* (electronic supplementary material, Results, section A). Our findings were not sensitive to these assumptions.

### (c) Training and evaluation of artificial neural networks

One way to analyse similarities and differences in colour from an animal's point of view is to employ colour space approaches to visualize chromatic information (see electronic supplementary material, Results, section C) (e.g. see [33]). However, in discriminating natural stimuli flies may use chromatic and/or achromatic information, so we employed ANNs in a manner analogous to photoreceptor-based models of colour choice that statistically relate calculated photoreceptor signals to behaviour [33]. In this context, ANNs have an advantage over traditional statistical approaches since they allow for parallel and nonlinear processing of photoreceptor signals closer to what we expect to occur in real nervous systems, so we suggest that they provide a useful tool with which to address such problems, especially where the dimensionality of colour vision is high.

We constructed ANNs to classify stimuli as ‘animal’ versus ‘leaf’ regardless of the illuminant (henceforth ‘animal-ANNs’), and ‘shaded’ versus ‘unshaded’ regardless of the stimulus type (henceforth ‘shaded-ANNs’), using the nnet package for R [25,42]. The ANNs were fully connected, feed-forward networks with three layers: an input layer, a hidden layer and an output layer. A single hidden layer was used to permit a nonlinear relationship between inputs and outputs without excessive complexity. Of principal interest were ANNs using the calculated excitations, *E*, of all five fly photoreceptors as their input layer. However, to better understand the mechanisms by which stimuli were classified, we also created ANNs with input from subsets of these photoreceptors. We created ANNs receiving four inputs to test the importance of excluding any single photoreceptor signal, two inputs to test which opponent comparisons could achieve effective classification, and single inputs to test whether classification could be achieved using achromatic information. In all cases, the hidden layer had three artificial neurons, and the output layer had one neuron with logistic activation function, which produced a binary outcome (i.e. 0 corresponding to ‘leaf’ or ‘unshaded’ classifications, and 1 corresponding to ‘animal’ or ‘shaded’ classifications; inflection point 0.5).

For each model type, we trained ANNs as follows. The stimulus dataset was randomly split to use 60% of the stimuli for training and 40% for testing. One-hundred ANNs were then trained on the training data using different randomly determined starting weights and maximum conditional likelihood to optimize performance. After training, maximum conditional likelihood was used to select the best model from the set of 100. This process was then repeated 20 times, each time using a new random assortment of the stimuli into training and testing subsets. Thus, we created an ensemble of 20 best-fitting ANNs selected from 2000 that were trained in total, with each of the selected ANNs trained using different subsets of our stimulus dataset. We deemed this sample adequate given that model accuracy varied little (see §3). After training, the selected ANNs were made to classify the 40% of stimuli that were set aside as test data for validation. To quantify classification accuracy, we recorded the proportion of classifications that were correct (i.e. true positives plus true negatives).

To evaluate how our 20 best-fitting ANNs processed photoreceptor signals to achieve accurate classification, we employed the clamping technique [43]. First, each ANN was made to classify the complete database of 288 stimuli and its accuracy was recorded. The performance of the network was then re-evaluated with one of its photoreceptor inputs clamped at the median value for that photoreceptor across all of the stimuli considered, meaning that it carried no useful information to aid in classification. During clamping, other photoreceptor inputs were presented to the network without any alteration. If clamping caused a notable reduction in the network's classification accuracy (using the level of random chance classification as a reference), then the clamped photoreceptor was deemed important. If a photoreceptor was deemed important, we further investigated how it contributed to the network's decision making. To do this, we identified the stimuli whose classifications changed as a result of the clamping procedure. For each of these stimuli, we calculated the difference between clamped and original excitation values for the photoreceptor in question. We then checked whether there was an association between the sign of these differences and the likelihood of reclassification as a particular stimulus class (e.g. whether increases in a given photoreceptor excitation value were associated with a greater proportion of stimuli being reclassified from ‘leaf’ to ‘animal’, versus ‘animal’ to ‘leaf’). The clamping procedure was applied to each photoreceptor in turn, allowing us to evaluate the way in which each individual photoreceptor contributed to classification. In addition, we provide connection weights for our 20 best-fitting ANNs of each type (electronic supplementary material, Results, section B).

### (d) Evaluating the appearance of blue stimuli

We next investigated how artificial, blue stimuli would appear to our trained ANNs to test the hypotheses that blue objects resemble animal hosts, and that blue objects resemble shaded resting places, in terms of the sensory information available to a fly. We obtained the reflectance spectra for a variety of blue fabrics used in biting fly control devices, or found to be highly attractive to biting flies in field studies (figure 1*d*; data from [3–5,24]). These included classic phthalogen blue-eyed cottons, which have very high attractiveness to biting flies and are traditionally used in control devices [3–5,13], and several modern blue polyesters produced by Vestergaard S.A. and used in biting fly control devices past and present [5,24]. Since black and violet (purple to a human eye) control devices are also effective for tsetse, we also obtained spectra for such fabrics [5,24]. Using these reflectance spectra, we calculated fly photoreceptor excitations as described above. We presented these data to our 20 best-fitting animal-ANNs and shaded-ANNs independently, and checked how often they elicited ‘animal’ and ‘shaded’ classifications, respectively. We expressed these data as a proportion for each artificial stimulus, wherein a value of zero indicated that all 20 models made ‘leaf’ or ‘unshaded’ classifications, and a value of one indicated that all 20 models made ‘animal’ or ‘shaded’ classifications. This proportion provided an estimate of the probability that an artificial stimulus would be mistaken for an animal or a shaded resting place, respectively, with high probabilities supporting the hypotheses that artificial and natural stimuli share salient features of the sensory information available to a fly.

## 3. Results

### (a) Performance of artificial neural networks

**Figure 2.**
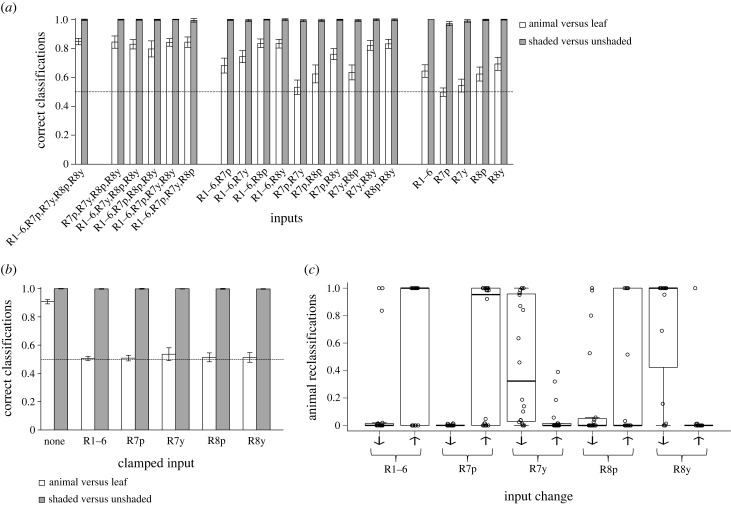
Evaluation of ANNs trained to distinguish ‘animal’ and ‘shaded’ stimuli. (*a*) The accuracy of trained ANNs in classifying a set of test stimuli not encountered during training, according to the number of photoreceptor excitation inputs they received. Animal-ANNs with more photoreceptor inputs had greater classification accuracy, but shaded-ANNs were similarly accurate regardless of their number of photoreceptor inputs. (*b*) The accuracy of trained ANNs in classifying the complete dataset of stimuli with the excitation value of individual photoreceptor inputs clamped to their median values. Data are for the full ANNs receiving all five photoreceptor inputs. Clamping any photoreceptor excitation value reduced the accuracy of animal-ANNs to random chance, indicating that all were important to accurate classification. However, clamping had no effect on the accuracy of shaded-ANNs, demonstrating that no single photoreceptor input was critical. (*c*) The contribution of photoreceptor excitation signals to classification by animal-ANNs. Plot considers only those stimuli whose classification changed as a result of clamping in (*b*). The proportion of these reclassifications in which the classification changed from ‘leaf’ to ‘animal’, rather than ‘animal’ to ‘leaf’, is shown, according to whether clamping increased or decreased the relevant photoreceptor excitation value for a given stimulus (direction of change in excitation values is indicated by an arrow). Increases in the excitation of blue-sensitive R1–6 (and UV-sensitive R7p) are associated with stimuli being reclassified as animals, and decreases are associated with reclassification as leaves. Increases in the excitation of green-sensitive R8y (and UV–blue-sensitive R7y) are associated with stimuli being reclassified as leaves, and decreases are associated with reclassification as animals. Each plot presents results for the best 20 ANNs of each type. In (*a*) and (*b*), these are collated as means and sample s.d. In (*c*), individual datapoints are plotted (circles), superimposed over boxplots showing 25th, 50th and 75th percentiles.

We trained animal-ANNs to classify ‘animal’ versus ‘leaf’ stimuli, and shaded-ANNs to classify ‘shaded’ versus ‘unshaded’ stimuli, using varying numbers of fly photoreceptor signals as inputs (figure 2*a*). Shaded-ANNs using all five fly photoreceptor signals as input classified test stimuli with very high accuracy (99.8% on average; figure 2*a*). Compared to this full model, shaded-ANNs receiving only one photoreceptor signal as input were similarly accurate in classifying stimuli (figure 2*a*). Thus, we conclude that shaded stimuli can be accurately classified using the achromatic information available in the response of any single photoreceptor type. Animal-ANNs using all five photoreceptor inputs were also effective, but had slightly lower accuracy in classifying test stimuli than the equivalent shaded-ANNs (84.7% on average; figure 2*a*). While the accuracy of some two-photoreceptor input animal-ANNs approached random chance, others maintained similar classification accuracy to the full model (figure 2*a*). Notably, three of the four best-performing animal-ANNs of this type received input from the green-sensitive R8y photoreceptor and various blue-sensitive photoreceptors (R1–6, R7y or R8p). The other received input from R1–6 and R8p, which are both blue-sensitive but have different *λ*_max_ values (figure 1*a*). The classification accuracy of animal-ANNs receiving a single photoreceptor signal input approached that of random chance, with most accuracy achieved by the ANN using the green-sensitive R8y signal as input. Thus, shaded stimuli could be classified accurately using the achromatic information available in the response of a single photoreceptor, but accurate animal classifications could only be achieved using the information available in the responses of more than one photoreceptor type.

### (b) Artificial neural network mechanisms

The mechanisms by which ANNs function can be difficult to determine from the connection weights of fitted models, but those for the full, five-input ANNs indicated the use of chromatic information (i.e. photoreceptor response comparison) by animal-ANNs, and achromatic information (i.e. photoreceptor response sums) by shaded-ANNs (electronic supplementary material, Results, section B). To interrogate these mechanisms, we challenged the full, five-input ANNs with the full dataset of photoreceptor excitation signals with the responses of a single photoreceptor clamped at its median value to negate its use in classification. The accuracy of shaded-ANNs was unaffected by clamping any single photoreceptor at its median value (figure 2*b*), indicating that these five channels of sensory information were redundant, and aligning with our previous finding that successful shade classification could be achieved on the basis of any single photoreceptor signal. Meanwhile, the accuracy of animal-ANNs fell to the level of random chance if any single photoreceptor response was clamped (figure 2*b*), indicating that classifications were made by these models using information available across all the photoreceptor signal inputs.

To understand the mechanisms used by animal-ANNs, we examined all classifications that changed as a result of clamping a given photoreceptor signal to its median value. We divided these according to whether clamping increased or decreased the original photoreceptor signal and examined the proportion of these reclassifications that changed from ‘leaf’ to ‘animal’, rather than ‘animal’ to ‘leaf’, in each case (figure 2*c*). Photoreceptor inputs had opposite effects on classification. For blue-sensitive photoreceptors R1–6 (and UV-sensitive R7p), increases in excitation tended to cause an increase in animal classifications, while decreases in excitation tended to cause an increase in leaf classifications. For green-sensitive photoreceptor R8y (and UV–blue-sensitive R7y), increases in excitation tended to cause an increase in leaf classifications, while decreases in excitation tended to cause an increase in animal classifications. There was variation in these effects across models, indicating that a variety of classification mechanisms could be effective (see also electronic supplementary material, Results, section A). In explanation of this, applying the same method to the best two-photoreceptor input animal-ANNs fitted above revealed that increased excitation in various blue-sensitive photoreceptors promoted animal classifications, while increased excitation in the green-sensitive R8y promoted leaf classifications (electronic supplementary material, Results, section B). Thus, these data provide evidence that chromatic information from the opponent processing of blue- and green-sensitive photoreceptors is an effective means of discriminating animals from leaves, but that a variety of different photoreceptors can be effective in these roles.

### (c) Classification of blue stimuli

Finally, we challenged our 20 best-fitting animal-ANNs and shaded-ANNs with photoreceptor excitations generated from the reflectance spectra of blue, black and violet fabrics that were investigated in previous studies of biting fly attraction (table 1). When viewed under open/cloudy illumination, these fabrics were very likely (0.75–1.00 probability) to be classified as animals. However, under such illumination, these fabrics were never classified as shaded. When viewed under woodland shade illumination, their probability of being classified as animals was little changed, but all fabrics were likely to be correctly classified as shaded (table 1). Thus, animal-ANNs often misclassified blue objects as animals, but shaded-ANNs did not misclassify them as shaded.

**Table 1.** The proportion of the 20 best ANNs of each type that classified fabrics used in biting fly control as ‘animals’ or as ‘shaded’. Proportions are for fabrics under open/cloudy illumination, with those for woodland shade illumination in brackets.

fabric	‘animal’ classifications	‘shaded’ classifications
phthalogen blue cotton [5]	1.00 (1.00)	0.00 (1.00)
phthalogen blue cotton [3]	0.75 (0.80)	0.00 (1.00)
blue fabric [4]	0.80 (0.85)	0.00 (1.00)
‘phthalogen blue’ polyester [5]	1.00 (1.00)	0.00 (1.00)
royal blue polyester [5]	1.00 (0.95)	0.00 (1.00)
typical blue polyester [24]	1.00 (1.00)	0.00 (0.70)
ZeroFly® blue polyester [24]	0.90 (0.95)	0.00 (1.00)
black cotton [5]	0.75 (0.70)	0.00 (1.00)
black polyester [5]	0.75 (0.60)	0.00 (1.00)
black cotton [24]	0.85 (0.70)	0.00 (1.00)
violet polyester [24]	1.00 (1.00)	0.00 (1.00)

## 4. Discussion

In this study, we used ANNs to understand the functional significance of attraction to blue stimuli in biting flies. We found that ANNs could be trained to discriminate animals from leaves, and shaded from unshaded surfaces, based upon the photoreceptor signals available to a fly. ANNs trained to discriminate animals from leaves did so based upon an opponent interaction between green- and blue-sensitive photoreceptors, and commonly misclassified artificial blue stimuli as animals. Meanwhile, ANNs trained to discriminate shaded from unshaded stimuli did so based upon achromatic information and never misclassified blue stimuli as shaded. Thus, blue–green opponency appears to be an effective mechanism for discriminating animal hosts from leaf backgrounds using the sensory information available to a fly, and through this mechanism blue objects resemble animal hosts.

We define chromatic information as information available through the comparison of photoreceptor responses [33], and our results provide several lines of evidence suggesting the importance of a blue–green comparison for a fly to discriminate animals from leaves (see also electronic supplementary material, Results, sections A–C). Animal-ANNs receiving input from a single photoreceptor type were considerably less accurate than many of those receiving input from multiple photoreceptors, indicating that achromatic information available in the responses of single photoreceptors was insufficient for this classification task. However, animal-ANNs receiving only two-photoreceptor inputs could perform similarly to the animal-ANN receiving all five, provided that those inputs were from green- and/or blue-sensitive photoreceptors. The connection weights of the animal-ANNs receiving all five photoreceptor inputs indicated photoreceptor response comparison at several levels within the trained networks (electronic supplementary material, Results, section B). By the clamping procedure, we showed that decreased excitation in green-sensitive R8y, and increased excitation in blue-sensitive photoreceptors, e.g. R1–6, was commonly associated with a greater likelihood of a stimulus being classified as ‘animal’, although there was variation in photoreceptor contributions across models (see also electronic supplementary material, Results, section A). The same principle was evident in the best two-photoreceptor input animal-ANNs, wherein accurate classification could be achieved by pairing the green-sensitive R8y with various different blue-sensitive photoreceptors, or two blue-sensitive photoreceptors with varying *λ*_max_ (electronic supplementary material, Results, section B). R1–6 have a broadband response and are usually associated with encoding luminance information, but it is now known that they contribute to colour vision in *Drosophila* [36]. R7y and R8y are often assumed to form an opponent pair [34,35], and the interaction of these photoreceptors has been implicated by photoreceptor-based models explaining tsetse catches at coloured targets in the field [14,21]. Our approach does not allow us to say which specific photoreceptor response comparisons are more probable in the nervous system of a real fly and instead our results suggest that several different comparisons can achieve comparable classification accuracy. We conclude that some form of blue–green opponency is sufficient to distinguish animal hosts from leaves using the sensory information available to a fly.

We define achromatic information as that present in individual photoreceptor responses, or sums of photoreceptor responses [33]. Shaded-ANNs discriminated shaded from unshaded stimuli based upon achromatic information since a shaded-ANN receiving any single photoreceptor input was just as accurate as a shaded-ANN receiving all five, connection weights in the full trained models indicated that photoreceptor responses were summed and not compared (electronic supplementary material, Results, section B), and in those full models, the five photoreceptor inputs provided redundant information as clamping had no effect on classification accuracy (see also electronic supplementary material, Results, section A). All photoreceptors performed similarly in this respect, and there was no special importance to blue-sensitive photoreceptors. This finding was unsurprising given the very large difference in intensity between woodland shade and open/cloudy illuminants, making low luminance by far the most obvious cue for discriminating shaded surfaces. On sunny days, as modelled here, the woodland shade illumination spectrum is relatively richer in shorter wavelengths versus the open/cloudy spectrum [19]. Therefore, subtle chromatic cues may have been available, but were not used by shaded-ANNs. In the real world, the illuminant spectra for shaded and open habitats are similar on overcast days [19], meaning that chromatic information might not always be available for this task.

Animal-ANNs commonly misclassified artificial blue stimuli as animals. Meanwhile, shaded-ANNs did not misclassify these stimuli as shaded. This suggests that, to a fly's eye view, blue objects are attractive because they share salient features of potential hosts, and not of shade. In support of this idea, tsetse caught at coloured targets in field experiments are relatively starved, indicating that they were seeking hosts [44]. Numerous previous field experiments have shown that biting flies are most attracted to targets that reflect blue wavelengths but not green or UV wavelengths [3–6]. In general, these properties agree with those used by our animal-ANNs, with the exception that the ANNs did not find any strong evidence for a negative influence of a UV-sensitive photoreceptor on animal classifications. This likely results from the fact that neither animal nor leaf stimuli reflect significant amounts of UV light, and thus this was not a feature that could be used to distinguish the two stimulus types investigated in this work. This fact might also explain why flies were not attracted to targets with significant UV reflectance in those field studies [3–6].

In constructing these models, we used a database of stimulus reflectance spectra intended to sample variation in the coloration of melanin-pigmented animal integuments and plant leaves. This was because spectra of each type conform to well-described patterns [18], so our intention was to represent natural variation in these patterns and consider the attraction of a generic biting fly towards a generic host in a generic environment. This meant that we did not specifically sample important animal hosts of any given biting fly species, nor leaves found within their natural environment, because the shapes of these spectra were represented by our stimulus set. In support of this point, spectra recorded for biting fly hosts such as cattle and deer have similar shapes to the animal spectra in our stimulus set [38].

Our study did not include sensory information beyond the responses of the five main classes of photoreceptor, and in doing so allowed us to focus solely on the importance of chromatic and achromatic visual information. However, information in other sensory modalities is undoubtedly important in host seeking. As described in our §1, natural spectra are said to belong to three classes, and those of animal melanin pigments are part of a large class of stimuli that includes many organic and inorganic surfaces, including tree bark, dead vegetation, rocks, and soil [18]. Thus, colour cannot on its own identify a potential host, and additional sensory information would be required, e.g. odour cues [1]. Interestingly, tsetse from savannah habitats are more responsive to odour than those from riverine habitats [12], and it is possible that odour cues are of greater relative importance when visual backgrounds contain a lot of dry, dead vegetation as opposed to green leaves.

One additional sensory cue that deserves special attention is polarization, because previous work on tabanids has shown that neural comparisons of photoreceptor responses confound colour and polarization cues, leading to the suggestion that the blue preference shown by biting flies is a by-product of a polarization sensing mechanism [17]. Polarization cues have been shown to be useful in the discrimination of bodies of water [45]. Tabanid flies seek bodies of water for egg laying, and some tsetse are associated with riverine habitats. It has also been proposed that polarization cues can be useful in discriminating sunlit animals with dark pelage from shaded vegetation backgrounds, because the former can appear highly polarizing [46]. However, it has been shown that the apparent high degree of polarization from dark surfaces is sometimes likely to be an artefact of the calculation procedures employed to quantify it [47], and our animal-ANNs were able to discriminate animals from leaves using colour, regardless of their illuminant, and without access to polarization cues. Furthermore, the dorsal margin of the housefly eye houses R7 and R8 photoreceptors specialized for the detection of polarized light and possessing the same spectral sensitivity [20], so evolution has equipped flies with an ability to detect polarization that is not confounded by colour. While our study cannot argue against the use of polarization cues by host-seeking tabanids, it has shown that a blue preference is functional in itself for identifying hosts, and while this might in some cases be enhanced by a co-occurring polarization sensitivity, it appears not to be a non-functional by-product of that mechanism.

Finally, biting flies are not alone among insects in being attracted to blue stimuli, as naïve pollinators commonly show such a preference [48,49], but obviously do not seek animal hosts. However, since flowers belong to the leaf contrast spectral class whose spectra follow no predictable pattern [18], the primary behavioural requirement of a naïve pollinator is to identify objects that are not foliage in order that those objects can be investigated and learnt associations with rewarding flower types formed. Therefore, this classification task is analogous to that investigated for biting flies in this study, and the similar behaviour likely occurs because blue–green opponency is an efficient way to segregate the reflectance spectra of natural objects using the sensory machinery of an insect eye.

## Data Availability

Data and R code are available at the Dryad Digital Repository: https://doi.org/10.5061/dryad.dr7sqvb3r [25]. Electronic supplementary material is available at FigShare [50].
